# Deep learning model for detecting high-grade dysplasia in colorectal adenomas

**DOI:** 10.1016/j.jpi.2025.100441

**Published:** 2025-04-05

**Authors:** Eric Steimetz, Zeliha Celen Simsek, Asmita Saha, Rong Xia, Raavi Gupta

**Affiliations:** a Department of Pathology, SUNY Downstate Health Sciences University, Brooklyn, NY, USA; b Department of Pathology, New York University School of Medicine, New York, NY, USA

**Keywords:** GI polyps, Tubular adenoma, High-grade dysplasia, Deep learning, Whole-slide images

## Abstract

**Objective:**

Early detection and removal of suspicious polyps during routine colonoscopies play an important role in reducing the risk of colorectal cancer. Patient management and follow-up are determined by the type of polyps removed and the degree of dysplasia present on histological evaluation. Whereas discerning between a benign polyp and a dysplastic one is a trivial task, distinguishing between tubular adenomas with low-grade dysplasia (LGD) and high-grade dysplasia (HGD) is a challenging task. In this study, we trained a deep learning model to distinguish between colorectal adenomas with LGD and HGD.

**Design:**

We retrieved 259 slides of adenomatous polyps taken between January 2011 and October 2024. Slides with HGD were reviewed by a subspecialty-trained GI pathologist. After excluding discordant and duplicate cases, 200 slides remained: 71 (35.5%) with HGD and 129 (64.5%) with LGD. The slides were divided into training (160 slides, 80%) and test (40 slides, 20%) sets. After patch generation and stain normalization, a ResNet34 model (pre-trained on ImageNet) was trained using 5-fold cross-validation. Slide classification was determined by aggregating patch-level predictions.

**Results:**

The model's slide-level prediction accuracy was 95.0%, correctly classifying all but 2 out of 40 slides. The model achieved an area under the receiver operating characteristic curve score of 0.981 and an F1 score of 0.923.

**Conclusions:**

This study demonstrates that deep learning models can accurately distinguish between colonic adenomas with LGD and HGD. Training on a larger dataset could increase the accuracy and generalizability of the model and should be a focus of further studies.

## Introduction

Colorectal cancer is the second most common cause of cancer death in the United States, with over 150,000 new diagnoses and 50,000 cancer deaths annually, making it a significant health concern.^1^^,^^2^ Current colorectal cancer prevention guidelines advise regular screening colonoscopies for individuals with average risk beginning at the age of 45, as the prevalence increases with age, and rescreening every 10 years if no dysplasia is detected.^2^ This regular screening is crucial in the early detection and subsequent reduction of colon cancer cases.^1^, ^2^, ^3^, ^4^

Most polyps removed during colonoscopies are benign, whereas 10–15% constitute adenomatous polyps, which have the potential of progressing to cancer over the span of about 10 years.^3^ Adenomas with high-grade dysplasia (HGD) are associated with an increased risk of future advanced neoplasia and greater risk of progression to cancer.^4^ Therefore, the official guidelines recommend that patients with HGD have a follow-up colonoscopy within a period of 1–3 years, whereas patients with low-grade dysplasia (LGD) can wait 5 years. Patients without any adenomas can wait 10 years between screenings.^2^^,^^5^

Distinguishing between benign (i.e., hyperplastic polyp) and adenomatous polyps is a trivial task for pathologists. However, detecting adenomas with HGD is challenging, as evident by the level of disagreement and interobserver variability among pathologists.^6^, ^7^, ^8^ Furthermore, oftentimes the HGD is confined to a small portion of a large polyp and can be easily missed, leading to a wrong diagnosis with adverse clinical consequences.^6^ Colonoscopies are a surveillance procedure that are routinely performed in a community setting, and the polyps removed are usually reviewed by general pathologists not specifically trained in GI pathology and might inadvertently miss a small focus with HGD. Having a screening tool that accurately identifies adenomatous polyps with HGD could ensure that those patients have an appropriate follow-up and can reduce the incidence and mortality associated with colorectal cancer.

Recent advancements in artificial intelligence (AI) and machine learning have shown potential in assisting pathologists across various domains. Deep learning, a subset of machine learning, has been used successfully in histopathology image classification tasks, such as nuclei segmentation,^9^^,^^10^ cancer identification^11^^,^^12^, hormonal status determination,^13^ and mitotic activity quantification.^14^^,^^15^ Notably, the FDA has recently approved the use of AI software to assist pathologists in reviewing prostate needle biopsies.

Convolutional neural networks (CNNs) are a class of deep learning algorithms that are designed to process visual input, learn spatial features, and recognize specific patterns. That has made them extremely popular in image analysis, as they can distinguish between different classes of images. For example, CNNs may be trained to identify the key features that distinguish a bird from a dog, or metastatic carcinoma from a benign lymph node. Residual neural network (ResNet) models are a family of CNNs with unique architecture that has proven to be highly accurate and efficient.^16^ The models are highly adaptable and once trained, a model can be easily modified to perform new tasks, such as distinguishing between different kinds of histopathological images, a process known as transfer learning.

In this study, we aim to train and evaluate the ability of a ResNet34 model to distinguish between low- and high-grade colorectal adenomas. Our goal is to see if the model can perform this task reliably, which can enhance the accuracy of the pathologist's diagnoses and improve patient outcomes.

## Methods

### Case selection

We searched our database and retrieved 259 H&E slides processed between January 2011 and October 2024 at a multispecialty hospital in Brooklyn, NY. Of those, 130 slides had a diagnosis of HGD and 129 LGD. The HGD slide was reviewed by a board-certified pathologist with subspecialty training in gastrointestinal pathology to confirm the diagnosis. There were 29 (22.3%) HGD slides which were discordant, and 30 (23.1%) were from different sections of the same polyp and were excluded from the study due to their morphological similarities, leaving a total of 200 slides: 71 (35.5%) with HGD and 129 (64.5%) with LGD.

### Image processing

Whole-slide scanning was performed by the Philips Ultra-Fast Scanner at 40×. Each slide was entirely split into 512 × 512 patches at 10× magnification. Patches containing less than 50% tissue were discarded, yielding 44,373 patches. The patches were then stain-normalized using the Vahadane method, a structure-preserving normalization technique,^17^ to ensure consistent staining across images.

### Data augmentation

To enhance the variability of the training dataset and prevent overfitting, we applied a series of data augmentation techniques to the patches. The transformations included random horizontal flipping (*P* = 0.5), random vertical flipping (*P* = 0.5), random rotation (±15°), and color jittering with adjustments in brightness (±0.1), contrast (±0.1), saturation (±0.1), and hue (±0.05). Additionally, we used random erasing with a probability of 0.1.

### Model training and validation

The slides were randomly split into a training set comprising 160 slides (80% of the total, with 103 (64.4%) classified as LGD and 57 (35.6%) as HGD) and test set containing 40 slides (20% of the total, with 26 (65%) LGD and 14 (35%) HGD). yielding 37,363 patches in the training set and 7010 patches in the test set. A 5-fold cross-validation was implemented on the training data, by further splitting it into training and validation subsets. To address the class imbalance observed in the dataset and prevent overfitting, we incorporated class weights into the loss function, ensuring that both cases with LGD and HGD were equally represented during training.

A PyTorch ResNet34 model (pre-trained on the ImageNet dataset) was adapted for binary classification. To reduce overfitting, the model's final block (the fully connected layer) was modified and enhanced with a custom sequential block that included a dropout layer (*P* = 0.3). The model was then trained on the normalized patches for 30 epochs. Training was conducted using a batch size of eight with four Nvidia A6000 GPUs. During the first 12 epochs, the model was optimized with the AdamW optimizer (initial learning rate: 5e-4) and a one-cycle learning rate scheduler. For the remaining 18 epochs, training continued with an SGD optimizer (initial learning rate: 5e-4) and a ReduceLROnPlateau scheduler.

### Data analysis

**Fig. 1:**
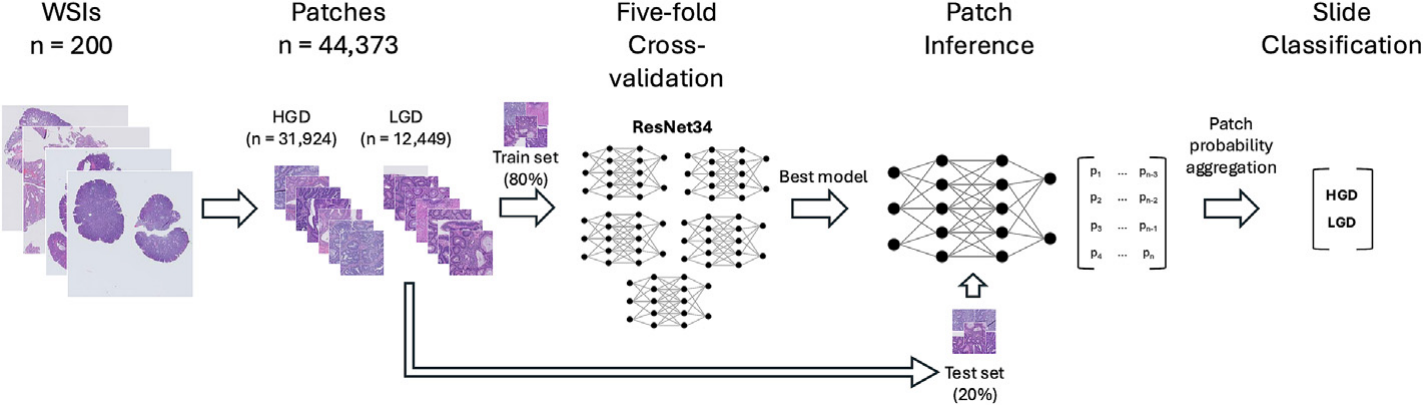
Overview of the key steps in the slide classification workflow.

Model performance was evaluated using the scikit-learn Python library. Metrics such as accuracy, area under the receiver operating characteristic curve (ROC AUC) score, and F1 score were computed to assess classification performance at both the patch and slide levels. Slide-level predictions were determined by aggregating the probabilities of all patches within a slide. The process is illustrated in Fig. 1.

## Results

Of the 44,373 patches included in the study, 37,363 were allocated to the training set and 7010 to the test set. The distribution of slides across LGD and HGD was 64.5%–35.5%, though the distribution across patches was 31,924 (71.9%) HGD and 12,449 (28.1%) LGD, respectively.

During 5-fold cross-validation, the model achieved an average patch-level accuracy of 80.6% (SD 3.52%), an F1 score of 0.855 (SD 0.0369) and an ROC AUC of 0.868 (SD 0.0464), indicating moderate consistency across folds. When patch probabilities were aggregated at the slide level, the model achieved an average accuracy of 91.3% (SD 6.37%), an F1 score of 0.866 (SD 0.103) and ROC AUC of 0.964 (SD 0.0214). The improvement in slide-level accuracy from patch-level accuracy suggests an effective aggregation of patch predictions. Consolidating these predictions across the entire slide reduces the influence of misclassified patches, resulting in a more reliable and accurate slide-level classification.

The best-performing model achieved a patch-level accuracy of 86.8%, an F1 score of 0.914 and an ROC AUC of 0.930, and a slide-level accuracy of 100%, an F1 score of 1.0 and an ROC AUC of 1.0 (Table 1). This model was used for classification of the test set.

**Table 1:** Fold metrics for patch-level and slide-level classification.

Fold	Patch-level			Slide-level		
	Accuracy	F1 Score	ROC AUC	Accuracy	F1 Score	ROC AUC
1	0.795	0.857	0.841	0.906	0.880	0.971
2	0.766	0.838	0.798	0.844	0.762	0.946
3	0.868	0.914	0.930	1.00	1.00	1.00
4	0.816	0.866	0.914	0.969	0.952	0.939
5	0.783	0.801	0.856	0.844	0.737	0.965
**Mean**	**0.806**	**0.855**	**0.868**	**0.913**	**0.866**	**0.964**

**Fig. 2:**
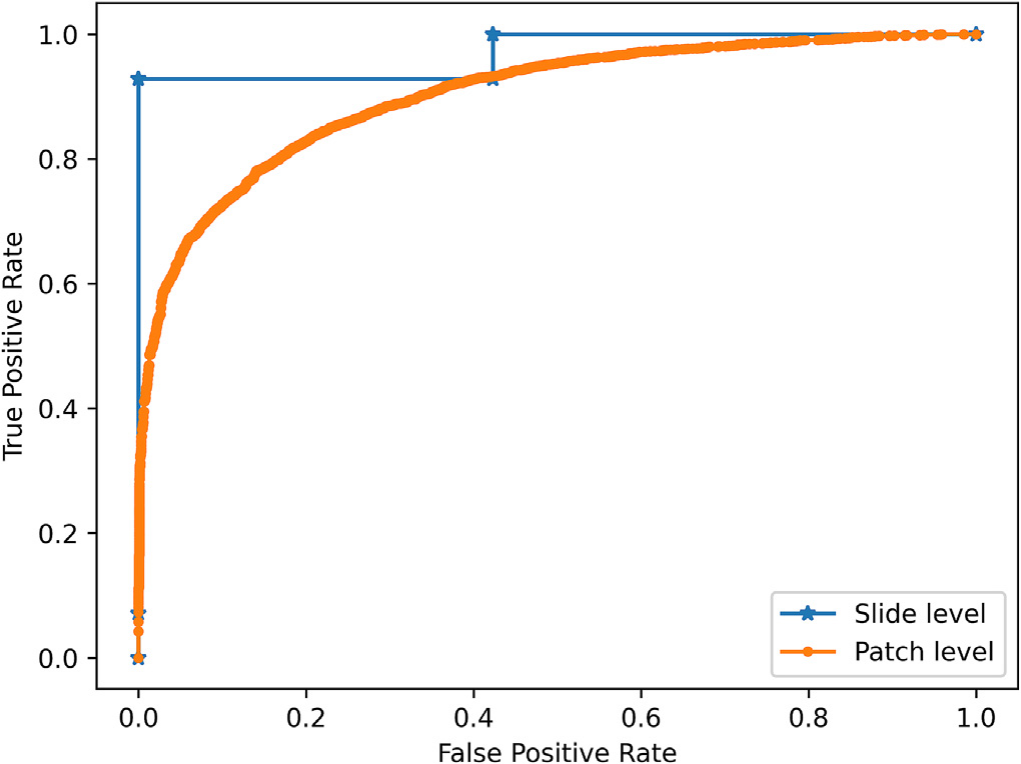
ROC curve illustrating the trained model's performance on the test set.

**Fig. 3:**
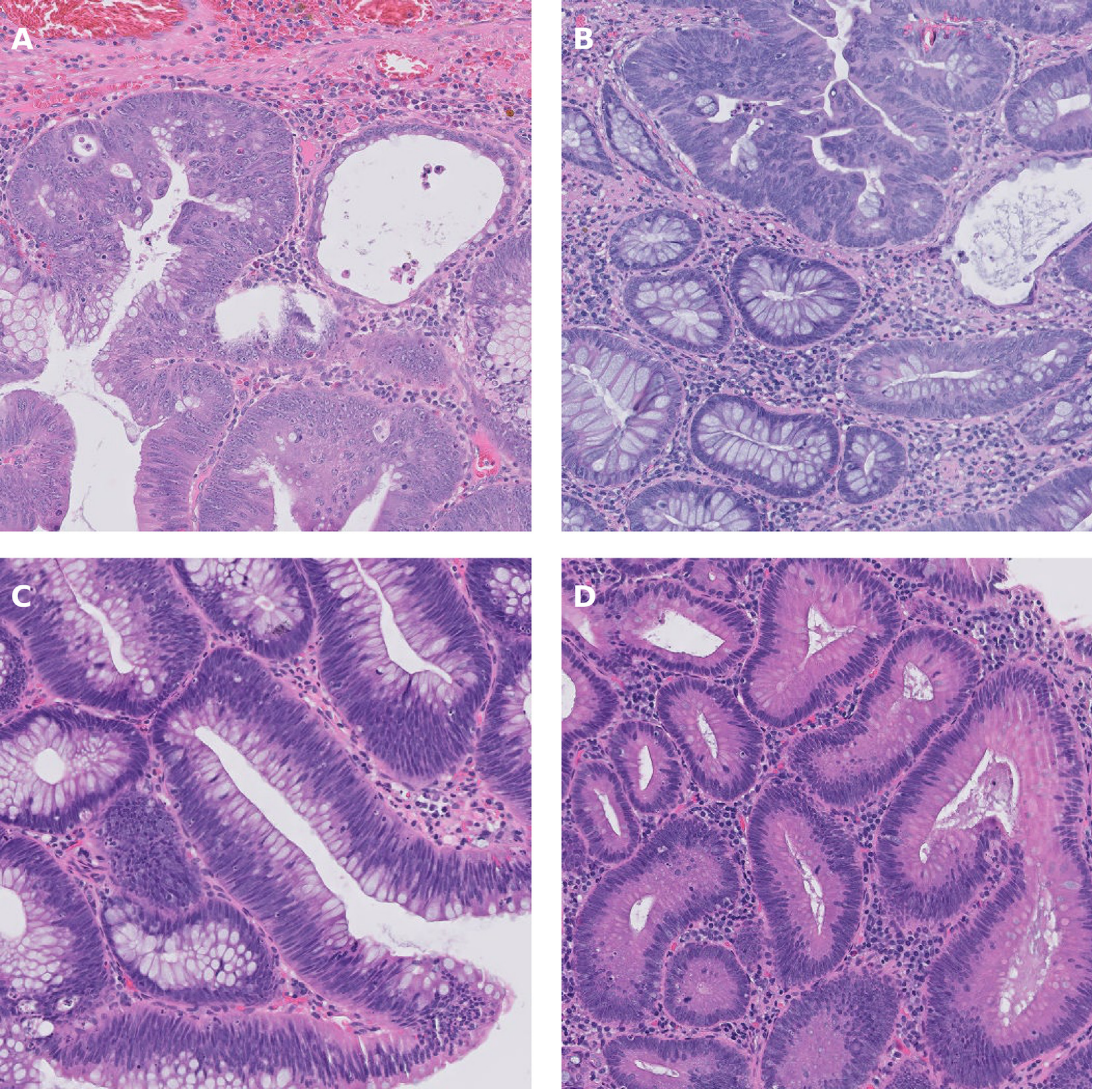
Examples of individual test set patches with their corresponding classification probabilities. Note that this may not reflect the final slide-level classification. (A) HGD correctly classified as HGD (HGD probability = 0.999). (B) HGD incorrectly classified as LGD (LGD probability = 0.772). (C) LGD correctly classified as LGD (LGD probability = 0.999). (D) LGD incorrectly classified as HGD (HGD probability = 0.999).

Slide-level classification of the test set, based on aggregate patch probabilities, resulted in an accuracy of 95%, correctly classifying all low-grade slides (26/26) and most high-grade slides (12/14) but misclassifying two of the high-grade cases as low-grade. The model's performance metrics at both the patch and slide level are summarized in Table 2. The ROC curve is shown in Fig. 2, and select patches are shown in Fig. 3.

**Table 2:** Model performance on test dataset**.**

	Accuracy	F1 Score	ROC AUC
Patch-level	0.792	0.830	0.901
Slide-level	0.950	0.923	0.970

## Discussion

The results of this study are encouraging, showcasing the potential of deep learning models to accurately distinguish between LGD and HGD in colorectal adenomas. The model achieved a prediction accuracy of 95%, correctly classifying all LGD slides. This highlights the model's utility in assisting pathologists with this challenging diagnostic task.

A notable aspect of this study is the simplicity of the model's implementation. Using a ResNet34 model (pre-trained on the ImageNet dataset), which is readily available via the PyTorch Python library, we were able to achieve a high level of accuracy. This approach demonstrates that such models can be easily integrated into existing digital pathology workflows. The results of this study suggest that this approach can be taken in other specimen types, training similar models to distinguish between cases that might pose a diagnostic challenge or pitfall.

**Fig. 4:**
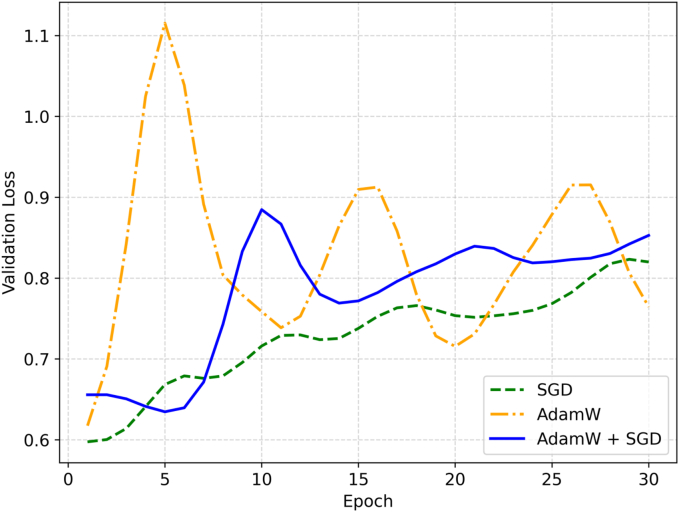
Comparison of validation loss across methods on Fold 2. The validation loss for AdamW (orange) fluctuates more significantly, whereas the AdamW + SGD combination (blue) and SGD (green) maintain a more stable trajectory. Lower validation loss indicates better generalization performance. (For interpretation of the references to color in this figure legend, the reader is referred to the web version of this article.)

Optimizers such as AdamW and SGD present distinct advantages and trade-offs in deep learning. Whereas AdamW offers a dynamic learning rate and faster convergence, it may exhibit overfitting and poor generalizability in some instances. Conversely, SGD is more stable but converges slowly and may get stuck in local minima or saddle points. To balance these trade-offs, we employed a hybrid approach, starting with AdamW for initial epochs followed by SGD, to prevent convergence to local minima. This approach achieved the faster initial convergence of AdamW while preserving the stability of SGD (Fig. 4). Whether this hybrid approach offers a consistent advantage over SGD alone requires further validation on larger datasets.

Similarly, while ResNet34 was chosen for its balance of accuracy, efficiency, and ease of implementation, this study does not aim to benchmark different architectures or determine the optimal model for this task. Future work could explore whether alternative architectures provide meaningful improvements.

Al models have shown great promise in the field of histopathology, performing tasks such as identifying regions suggestive of malignancy,^18^^,^^19^ narrowing the differential diagnosis of unknown primary tumors,^12^ predicting tumor genomics,^20^ and grading kidney disease.^21^ Rather than striving to replace pathologists, these tools can help them make better diagnoses and improve patient outcomes. For example, by having an AI model predict the top differentials for a small biopsy from a mass with an unknown primary malignancy, the pathologist would only order a limited number of ancillary immunohistochemistry stains, preserving the rest of the tissue for molecular or genomic testing. Another use case is histological grading of kidney disease, where AI models could potentially quantify the degree of important elements such as sclerosis, reducing the need for ancillary stains. The results of our study should be viewed in this context. Training a model to accurately classify and recognize every possible pathology that can be found in a colonic polyp (including for example, neuroendocrine hyperplasia or metastatic carcinoma) would require enormous resources that are beyond the reach of many pathology practices. However, deploying a model that can detect adenomas with HGD, a diagnosis that could be easily overlooked, could function as a valuable quality assurance tool and potentially improve patient outcomes.^22^

### Limitation

Our studies have several limitations. First, it was performed on a limited dataset of only 200 cases. Due to the relative rarity of the HGD cases, collecting a larger dataset was challenging. Furthermore, the test set contained only 14 HGD cases, meaning that although the overall slide-level accuracy was 95%, approximately 14% of HGD cases were misclassified, potentially affecting the reliability of this estimate. Second, the cases were all collected from a single institution. Tissue processing, staining, and slide preparation methods vary across institutions and may introduce subtle differences that could affect the model's performance.^23^^,^^24^ Although the slides were stain-normalized, it may not account for all the variability the model may encounter. Furthermore, a prospective study is needed to assess the feasibility of deploying such models in real-world settings, as different preparations techniques, such as using a different whole-slide scanner, may induce a data shift and degradation in performance.^24^

Lastly, we implemented patch probability aggregation, a straightforward method, for slide classification. Our analysis showed that this approach yielded results nearly identical to majority voting, with no impact on slide classification. Other techniques, such as threshold- or attention-based scoring have been described in the literature and warrant further investigation to assess their potential for improving classification accuracy.

## Conclusions

This study demonstrates that deep learning models can accurately distinguish between colonic adenomas with LGD and HGD. Such models could potentially be deployed in the background to screen for potential HGD cases and function as a quality assurance measure. Further prospective studies on larger datasets can potentially increase the accuracy and generalizability of these models.

## Declaration of generative AI and AI-assisted technologies in the writing process

During the preparation of this work the author(s) used GPT-4 in order to improve language and readability. After using this tool/service, the author(s) reviewed and edited the content as needed and take(s) full responsibility for the content of the publication.

## Declaration of competing interest

The authors declare that they have no known competing financial interests or personal relationships that could have appeared to influence the work reported in this article.
